# Efficacy and Safety of Botulinum Toxin Type A in Treating Patients of Advanced Age with Idiopathic Trigeminal Neuralgia

**DOI:** 10.1155/2018/7365148

**Published:** 2018-04-05

**Authors:** Jing Liu, Ying-Ying Xu, Qi-Lin Zhang, Wei-Feng Luo

**Affiliations:** ^1^Department of Neurology and Suzhou Clinical Research Center of Neurological Disease, The Second Affiliated Hospital of Soochow University, Suzhou 215004, China; ^2^Jiangsu Key Laboratory of Translational Research and Therapy for Neuro-Psychiatric-Diseases, Soochow University, Suzhou, Jiangsu 215123, China

## Abstract

**Objective:**

To assess the therapeutic efficacy and safety of botulinum toxin type A (BTX-A) for treating idiopathic trigeminal neuralgia (ITN) in patients ≥80 years old.

**Methods:**

Selected patients (*n*=43) with ITN, recruited from the neurology clinic and inpatient department of the Second Affiliated Hospital of Soochow University between August 2008 and February 2014, were grouped by age, one subset (*n*=14) ≥80 years old and another (*n*=29) <60 years old. Each group scored similarly in degrees of pain registered by the visual analogue scale (VAS). Dosing, efficacy, and safety of BTX-A injections were compared by group.

**Results:**

Mean dosages of BTX-A were 91.3 ± 25.6 U and 71.8 ± 33.1 U in older and younger patients, respectively (*t*=1.930,  *p*=0.061). The median of the VAS score in older patients at baseline (8.5) declined significantly at 1 month after treatment (4.5) (*p*=0.007), as did that of younger patients (8.0 and 5.0, resp.) (*p*=0.001). The median of the D values of the VAS scores did not differ significantly by group (older, 2.5; younger, 0; *Z*=−1.073, *p*=0.283). Two patients in each group developed minor transient side effects (*p*=0.825). Adverse reactions in both groups were mild, resolving spontaneously within 3 weeks.

**Conclusions:**

BTX-A is effective and safe in treating patients of advanced age (≥80 years old) with ITN, at dosages comparable to those used in much younger counterparts (<60 years old).

## 1. Introduction

Trigeminal neuralgia (TN) is characterized by paroxysms of intense, stabbing pain in the distribution of mandibular and maxillary divisions (rarely, the ophthalmic division) of the fifth cranial nerve. TN is one of the most common neurological pains involving the orofacial region, which generally has the most intensive type of pain [1]. According to epidemiologic studies, approximately 4–28.9/100,000 persons worldwide have experienced TN. It typically affects the elderly (1 in 25,000 of the population), with the most frequently reported cause being neurovascular compression [2, 3]. The morbidity of idiopathic trigeminal neuralgia increases with age, patients ≥80 years old account for a large proportion of TN sufferers [4]. Usually, TN patients are first treated with pharmacological agents [5]. The pain can be readily managed with medication in approximately 80% of patients. The first-line treatment is carbamazepine, which can relieve most of the observed symptoms. Other drugs, including oxcarbazepine, phenytoin, baclofen, lamotrigine, gabapentin, and sodium valproate, are also efficient in reducing the signs-symptoms of TN in most patients. Many drugs used in the treatment of TN are associated with several side effects, such as dizziness, lethargy, lack of fatigue, nausea, vomiting, occasional granulocyte reduction, reversible thrombocytopenia, and even induced aplastic anemia and toxic hepatitis. Considering insufficient effect or unacceptable side effects of pharmacological treatment, surgical treatment becomes an option. Several surgical approaches used to relieve the pain due to TN include neurectomy of the trigeminal nerve branches outside the skull, percutaneous radiofrequency thermal rhizotomy, percutaneous ablation that creates the trigeminal nerve or trigeminal ganglion lesions with heat, percutaneous retrogasserian glycerol rhizotomy, injection of glycerol into the trigeminal cistern, physical compression, trigeminal ganglion balloon microcompression, alcohol injections, botulinum toxin injection, cryotherapy, and gamma-knife radiosurgery (GKRS). Some of the surgical procedures may contribute to some complications [5], such as hearing loss, facial paresthesia, hypoesthesia, masseter weakness and paralysis, keratitis, transient paralysis of cranial nerves III and VI including diminished corneal reflex, dysesthesia, and anesthesia dolorosa, even an immediate complete loss of vision in one eye after trigeminal radiofrequency rhizotomy due to acute traumatic optic neuropathy.

In 2002, Micheli et al. first reported that BTX-A injection can relieve TN [4], supported by subsequent positive reports [6], and many studies have shown that botulinum toxin type A (BTX-A) may be effective and safe as treatment of TN [7–10]. The common side effects of botulinum toxin treatment on TN are dema/hematoma and facial asymmetry which include the muscle relaxation, distortion of commissure, and the ptosis of the eyelids at the site of injection. All these side effects were mild and automatically disappeared without any further treatment. No systemic side effects were observed [11, 12]. In February 2008, the U.S. Food and Drug Administration notified the public that Botox and Botox Cosmetic (botulinum toxin type A) and Myobloc (botulinum toxin type B) have been linked in some cases to adverse reactions, including respiratory failure and death, following treatment of a variety of conditions using a wide range of doses. The adverse reactions appear to be related to the spread of the toxin to areas distant from the site of injection, and mimic symptoms of botulism, which may include difficulty swallowing, weakness, and breathing problems. So the general fragility and often coexistent diseases of older patients imply perhaps greater susceptibility to side effects and needed reduction of BTX-A dosage. This research objective was to assess the therapeutic efficacy and safety of BTX-A in treating patients of advanced age (≥80 years old) with idiopathic trigeminal neuralgia (ITN).

## 2. Materials and Methods

### 2.1. Subjects

Eligible patents were recruited between August 2008 and February 2014 from the neurology clinic and the inpatient department of the Second Affiliated Hospital of Soochow University and were approved by the hospital ethic committee. Inclusion criteria were as follows: (i) diagnosis of classic ITN, as stipulated in the current version of the International Classification of Headache Disorders (ICHD-II); (ii) no prior exposure to BTX-A treatment; and (iii) failure of accepted medical and surgical interventions. Any conditions potentially heightening the risk of patient exposure to BTX-A (e.g., myasthenia gravis and motor neuron disease) or lack of pertinent medical information were grounds for exclusion.

### 2.2. General Information and Grouping

The study choose two groups of trigeminal neuralgia patients, one comprised of patients ≥80 years old (*n*=14) and another of patients <60 years old (*n*=29). At baseline (prior to treatment), the median pain scores by the visual analogue scale (VAS) in both older and younger patient groups were 8.5 and 8.0, respectively, showing no significant difference (*Z*=−1.411,  *p*=0.158). In the older patient group (male, 4; female, 10), each patient suffered from hypertension, diabetes mellitus, and cardiac insufficiency, and two displayed hepatic compromise. The age range was 80–90 years (average, 82.6 ± 2.9 years). In the younger group of patients (male, 10; female, 19), one suffered from hypertension. Ages ranged from 34 to 59 years (average, 49.5 ± 6.3 years). Gender ratios of the two groups were similar (*p*=0.968), but the incidence of coexistent diseases in the older (versus younger) age group was significantly higher (*p*=0.005) (Table 1).

### 2.3. Treatment

BTX-A (100 U clostridium botulinum type A neurotoxin complex, 5 mg gelatin, 25 mg dextran, and 25 mg saccharose) was commercially procured (Lanzhou Institute of Biological Products, Lanzhou, China) and diluted to 25 U/mL for treatments, drawing 1-2 mL from vials for injection. Administration was guided by each patient's perceived pain and trigger zones, delivering BTX-A intradermally and/or submucosally via 1 mL syringe. The total dosages delivered varied, ranging from 30 to 200 U.

### 2.4. Measures

Pain severity was assessed through patient input (interview or telephone), using the visual analogue scale (VAS). Patient examinations were conducted at baseline and at 1 month after treatment, recording related side effects.

## 3. Statistical Analysis

All analytics assumed an intent-to-treat basis, using two-sided testing. If obeying normal distribution, data were assessed using mean ± SD. And between-group comparisons were evaluated by means of *t*-test, comparing incidences via the chi-square test. If not, data were assessed using median values and the rank sum test was used. Standard software (SPSS v17.0; SPSS Inc. (IBM), Chicago, IL, USA) was engaged for statistical computations, setting significance at *p* < 0.05.

## 4. Results

The dosages of BTX-A were 45 to 150 U in the older group and 30 to 200 U in the younger group. Mean BTX-A dosages of 91.3 ± 25.6 U and 71.8 ± 33.1 U were administered in older and younger patient groups, respectively (*t*=1.930,  *p*=0.061). Median VAS scores 1 month after treatment in older (4.5) and in younger (4) patients were significantly lower than corresponding baseline values; and D values of VAS scores did not differ significantly by group (older, 2.5; younger, 0; *Z*=−1.073, *p*=0.283), reflecting similar group therapeutic outcomes. Transient minor side effects developed in two older patients (whole-body discomfort in one, mild left eye ptosis, and slight oral deviation/drooling in the other) and in two younger patients (mild facial paralysis comes in one and moderate facial paralysis comes in the other). Thus, the incidences of side effects did not differ significantly in these groups (*p*=0.825), and all events resolved spontaneously within 3 weeks (Tables 1 and 2).

## 5. Discussion

TN is further categorized as idiopathic (ITN) or secondary type. ITN occurs in the absence of neurologic signs or organic lesions, whereas secondary TN is due to tumors, multiple sclerosis, small infarcts, or angiomas arising in the pons or medulla. In the patients of this study recruited, secondary TN was excluded, given a lack of clinical symptoms, physical evidence, and imaging abnormalities.

Although the mechanisms involved in TN remain unclear, there are three major hypotheses for its development [13, 14]: (1) the trigeminal nerve compression, (2) irritative lesions impacting the thalamic corticospinal nucleus of the trigeminal nerve, and (3) short-circuiting of the trigeminal nerve. Many treatments available for TN include medical agents, nerve blocks, surgical interventions, and stereotactic radiotherapy. Antiepileptic drugs, especially carbamazepine, are still the first-line medical treatment, and long-term antiepileptic medication is often required. However, the risk of side effects, such as nausea, dizziness, dystaxia, hepatic insufficiency, and leukopenia, increases with prolonged use [15]. Microvascular decompression is the most widely adopted surgical treatment worldwide, but the recurrence rate is 20–30%, and a host of potential complications may develop during and after surgery [16]. In light of current evidence, it seems fair to argue for a safer, better tolerated, and more efficacious treatment.

BTX-A is one of the most potent neurotoxins, whether natural or synthetic. It prevents axonal release of acetylcholine (Ach), thus blocking neuromuscular transmission and producing muscle relaxation. Common clinical uses include blepharospasm, hemifacial spasm, dystonia, and cosmetic imperfections [17]. BTX-A also readily blocks cholinergic synapses in salivary and sweat glands and is therefore useful in suppressing glandular hyperactivity. In 2002, Micheli et al. was the first to identify BTX-A as an agent for TN relief [4]. Subsequent studies also have shown the benefits of BTX-A in treatment of pain (including TN) [18]. BTX-A offers an effective means of treating TN that is increasingly gaining attention, rather than confronting the side effects of medical or surgical treatments.

The precise mechanism of action for BTX-A in pain relief is not well defined but may be multifactorial. When injected directly into contracting muscles, BTX-A binds to presynaptic nerve terminals and becomes internalized, preventing exocytosis of the neurotransmitter acetylcholine (Ach) at neuromuscular junctions. BTX-A may also exert peripheral neurovascular activity by inhibiting release of various neurotransmitters, such as substance P, neurokinin A, calcitonin gene related peptide (CGRP), and enteral polypeptide [18]. These transmitters act on blood vessels and glutamate, relieving pain by inhibiting neurogenic inflammation and reducing afferent nerve impulses [19, 20]. What is more, BTX-A exerts an antinociceptive function, directly modulating central sensitization by inhibiting excessive expression of TRPA1, TRPV1, and TRPV2 in the spinal trigeminal nucleus [21, 22]. Finally, BTX-A or its metabolite likely reduces sympathetic nerve transmissions to curb suppression of Renshaw cells within inhibitory intermediate neurons, acting upon the spinal cord indirectly to alleviate pain [23]. A new study has just revealed that the antinociceptive effects of BTX-A are conferred by inhibiting Nav1.7 upregulation in the trigeminal ganglion [24]. Thus, such effects are not confined to the neuromuscular junction, acting as well on central nerve structures (i.e., trigeminal ganglion, trigeminal nerve ridge nucleus, or spinal cord). Further research is needed to explore the pathways by which BTX-A relieves the pain of TN.

A recently published meta-analysis has concluded that BTX-A may be an effective and safe treatment option for patients with TN, yielding on average a 29.8% reduction in paroxysms per day [11]. In our preliminary study, it was found that pain relief peaked 1 month after local injection of BTX-A [10]. Hence, this study established a 1-month monitoring interval. In this study, VAS scores were performed again in patients with primary trigeminal neuralgia after injection of BTX-A for 1 month. The study found VAS scores had significantly lowered than those before treatment in both groups, and this suggested that BTX-A was effective for both younger and elderly. Meanwhile, the D values of VAS scores did not differ significantly between the two groups, indicating the similar curative effect for both groups.

In addition to the volumes of injected BTX-A, routes of injection, injection frequencies, and injection sites have varied among studies. As little as 25 U of BTX-A was administered according to Zhang et al. [7], compared with a maximum of 100 U given by Shehata et al. [9]. Generally, BTX-A is delivered via subcutaneous or intradermal route [11]. In patients of advanced age, heart disease and compromised respiratory function often coexisted, but the need for reduced dosages of BTX in older patients or their vulnerability to its side effects has not been addressed. The injection sites selected in this study were perceived pain and trigger zones. The injection dosage was determined according to the range of pain, and a multipoint injection method was adopted. In this study, the volumes of injected BTX-A ranged from 30 to 200 U, delivered through intradermal and/or submucosal routes. The dosage of BTX-A is 45 to 150 U in the older group and 30 to 200 U in the younger group. Though the mean BTX-A dosages of the older group was little higher than those of the younger group, no significant between-group difference in BTX-A dosage materialized, and the corresponding incidences of side effects did not differ significantly. Two groups improved pain fairly, prompts that both small dose and large dose may have obvious curative effects. This finding is consistent with the conclusion of Zhang et al. [7], which suggested that BTX-A injection in TN was safe and efficient, and low dose (25 U) and high dose (75 U) were similar in efficacy in short term.

In terms of safety, the two reported side effects of BTX-A injection have been facial asymmetry and injection-site edema/hematoma, both of which prove tolerable and transitory in nature [11]. In total, occurrences of facial asymmetry and edema/hematoma following BTX-A injection have ranged from 2 to 5% and 1-2%, respectively. Facial asymmetry typically requires 5–7 weeks for resolution, whereas edema/hematoma persists for just 5-6 days. Rates of coexistent diseases, in this study, such as hypertension, diabetes mellitus, renal insufficiency, and cardiac insufficiency, were significantly higher in the older patient group by comparison, but as in the younger group, only two patients developed transient minor side effects (i.e., whole-body discomfort, mild ptosis, and oral deviation/drooling). All resolved within 3 weeks and without serious adverse reactions. Consequently, in extremely older patients afflicted with various medical conditions dose reductions seem unwarranted. Of course, it also seemed that the dosages of BTX-A in advanced age patients with side effects were 100 U and 150 U and in the younger patients were 30 U and 75 U. It seemed that the more the dosage in the older group, the greater the likelihood of side effects is. Therefore, it is suggested that it should be more carefully followed to see whether the patients have side effects after the treatment of elderly patients using a large dose of BTX-A.

Overall, BTX-A is an effective and safe treatment for ITN in patients of advanced age (≥80 years old), at dosages similar to those used in much younger counterparts (<60 years old). However, this study was limited to 14 older subjects, with no placebo group as reference, calling for more extensive randomized controlled trials.

## Figures and Tables

**Table 1:** Comparison of patient parameters in older (≥80 years old) and younger (<60 years old) BTX-A therapeutic groups.

Clinical index	Older group (*n*=14)	Younger group (*n*=29)	*p* value
Average age (years)	82.6 ± 2.9	49.5 ± 6.3	0.000
Gender (male/female)	4/10	10/19	0.968
Coexistent diseases (with/without)	6/8	1/28	0.005
Therapeutic doses (U)	91.3 ± 25.6	71.8 ± 33.1	0.061
D value of VAS (media, before and after treatment)	2.5	0	0.283
Side effect (total count)	2^a^	2^b^	0.825

^a^Whole-body discomfort in one; mild left eye ptosis and slight oral deviation/drooling in the other; ^b^Mild facial paralysis in one; moderate facial paralysis in the other; BTX-A: botulinum toxin type A; VAS: visual analogue scale.

**Table 2:** Comparison of VAS scores in older (≥80 years old) and younger (<60 years old) BTX-A therapeutic groups.

	VAS score before treatment (median)	VAS score after treatment (median)	*Z*	*p*_1_ value
Older group (*n*=14)	8.5	4.5	−2.680	0.007
Younger group (*n*=29)	8.0	5.0	−3.360	0.001
*Z*	−1.411	−0.040	—	—
*p*_2_ value	0.158	0.968	—	—

*p*_1_: comparison of VAS scores between before treatment and after treatment; *p*_2_: comparison of VAS scores between older group and younger group.

## Abbreviations

BTX-A: Botulinum toxin type A
ITN: Idiopathic trigeminal neuralgia
VAS: Visual analogue scale
TN: Trigeminal neuralgia
Ach: Acetylcholine
CGRP: Calcitonin gene related peptide
TRP: Transient receptor potential
Nav: Voltage gate sodium channel.
